# Eruptive Pruritic Papular Porokeratosis in a 77-Year-Old Male: A Rare Case

**DOI:** 10.7759/cureus.91086

**Published:** 2025-08-27

**Authors:** Richard L Dookie, Paul Subrt, Jaime Tschen

**Affiliations:** 1 Clinical Documentation, Landmark Medical Center, Woonsocket, USA; 2 Dermatology, Katy Westside Dermatology, Houston, USA; 3 Dermatology, St. Joseph Dermatopathology, Houston, USA

**Keywords:** cornoid lamella, dermato-pathology, disseminated superficial porokeratosis, eruptive pruritic papular porokeratosis (eppp), inflammatory dermatoses, pathology derm, pruritic lesions

## Abstract

Eruptive pruritic papular porokeratosis (EPPP) is a rare inflammatory variant of disseminated superficial porokeratosis characterized by the sudden onset of pruritic papules, primarily affecting the extremities and trunk. We present the case of a 77-year-old male who developed EPPP without any underlying malignancy or immunosuppression. Histopathology confirmed EPPP, and the patient underwent treatment with topical lovastatin 2% ointment, followed by topical calcipotriene. This report highlights the clinical presentation, diagnostic workup, therapeutic interventions, and emerging insights into the management of this uncommon condition.

## Introduction

Porokeratosis represents a heterogenous group of keratinization disorders defined histologically by the presence of a cornoid lamella - an abnormal column of parakeratosis overlying an absent granular cell layer [1]. Common clinical variants of porokeratosis include disseminated superficial actinic porokeratosis (DSAP), disseminated superficial porokeratosis (DSP), linear porokeratosis, and porokeratosis of Mibelli. Clinically, all variants feature a central area of atrophy encircled by a sharply defined, raised hyperkeratotic border [1].

Eruptive pruritic papular porokeratosis (EPPP) is a rare inflammatory variant of DSP. It was first described by Kanzaki et al. in 1992 as a new clinical variant of DSP distinguished by acute onset of pruritic inflammation superimposed on traditional porokeratosis lesions [2]. It was later termed “inflammatory DSP” by Tanaka et al. in 1995 [3]. EPPP typically follows three phases: a prolonged asymptomatic rash, an eruptive pruritic phase, and gradual resolution [2,3]. While the exact etiopathogenesis remains uncertain, contributing factors include ultraviolet radiation, immunosuppression, infections, neoplastic processes, and pharmacologic triggers [4,5]. Associations with type 2 diabetes mellitus and corticosteroid-induced immunosuppression have also been documented.

With fewer than 50 cases reported in the literature to date, EPPP predominantly affects older adults, with a median age of onset around 65 years and a slight male predominance. The condition appears to have no clear racial or geographic predilection and is typically sporadic, though familial clustering has occasionally been observed in other porokeratosis subtypes [5].

Histologic findings often overlap with DSP and other forms of porokeratosis, but inflammatory infiltrate and interface dermatitis offer distinguishing features [5]. In rare cases, DSP has been associated with secondary cutaneous amyloidosis, with amyloid deposits observed in the dermis or confined to the lesional epidermis, suggesting an epidermal origin of the amyloid. While this phenomenon has not yet been clearly reported in EPPP, it may represent a potential overlap worthy of further study. Additionally, recent literature has explored emerging associations between EPPP and viral infections, particularly SARS-CoV-2, suggesting a potential immunologic trigger in genetically predisposed individuals [5].

Spontaneous resolution may occur in EPPP, but symptom severity often necessitates intervention. Management remains challenging, with inconsistent responses to conventional therapies. Topical corticosteroids, retinoids, and cryotherapy have been used with variable success. More recently, emerging therapies such as JAK inhibitors, omalizumab, and topical calcipotriene (a vitamin D analog with immunomodulatory properties) have shown promise in refractory or severe cases [6,7].

Correctly identifying EPPP from other forms of porokeratosis is essential. It allows clinicians to apply a more precise diagnostic workup, emphasizing internal malignancy screening and to adopt a targeted anti-inflammatory treatment approach, marking a significant shift from the management of traditional DSP variants [8,9]. Despite advances, EPPP remains poorly understood due to its rarity and the limited number of documented cases. Continued case-based reporting and a deeper understanding of its pathogenesis are critical for improving diagnostic accuracy, optimizing treatment strategies, and clarifying its clinical course.

## Case presentation

Patient information

A 77-year-old white male presented to the dermatology clinic on March 19, 2025, with a six-month history of a pruritic skin eruption localized to both lower legs. He described a sudden onset with progressive increase in the number of lesions over time. He denied any antecedent viral infections, medication use, or systemic symptoms such as fever, weight loss, or night sweats. Apart from the skin lesions, he was in good general health with no significant medical history. He was not on any medications and had no known history of allergies, autoimmune disease, prior dermatologic conditions, immunosuppression, or malignancy. His surgical, family, social, and travel histories were non-contributory.

Clinical findings

**Figure 1:**
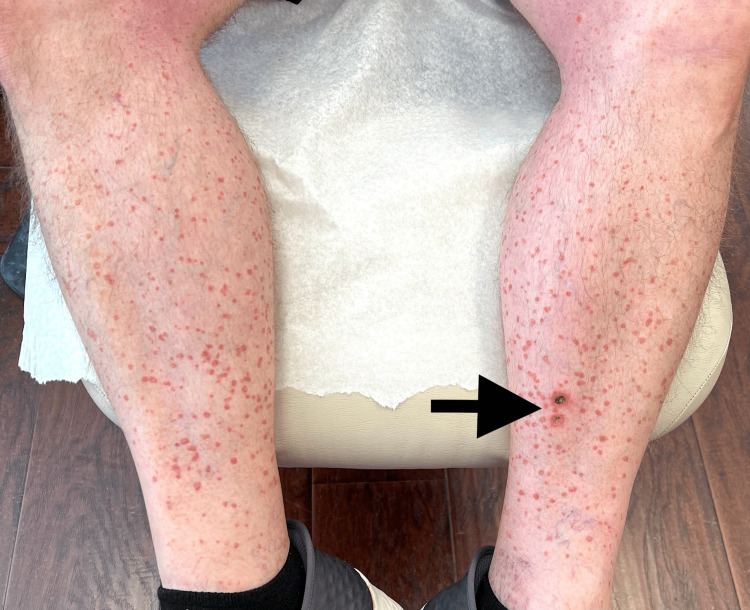
Clinical image of eruptive pruritic papular porokeratosis Widespread monotonous small, annular, erythematous pruritic macules and papules distributed bilaterally on the lower limbs. The two shave biopsy sites (arrow) are present in the distal and medial surface of the left shin.

**Figure 2:**
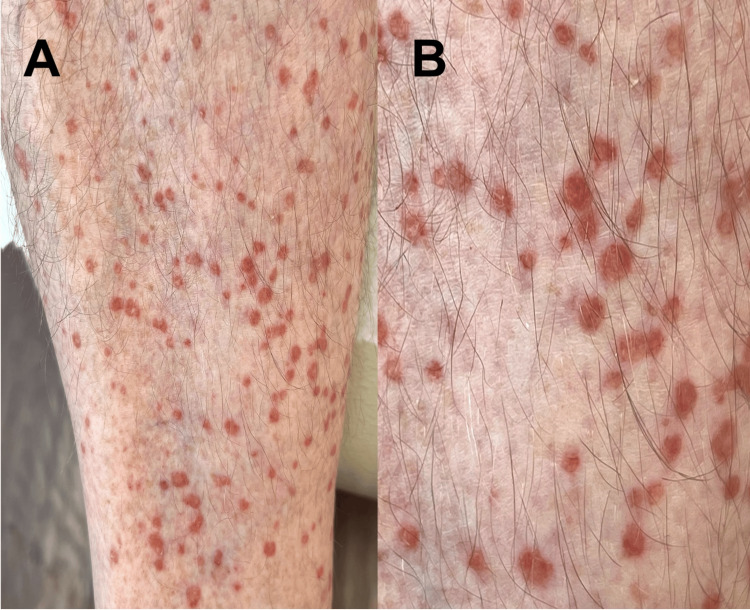
Clinical image of eruptive pruritic papular porokeratosis (A) Numerous 3- to 5-mm reddish-brown, annular, flat-topped papules on the lower extremities. (B) Each papule is bordered by a peripheral hyperkeratotic ridge consistent with a cornoid lamella.

On physical examination, numerous 3- to 5-mm reddish-brown, flat-topped papules were noted bilaterally on the lower extremities, and many of the lesions exhibited a subtle raised border (Figures 1, 2). There were no lesions on the trunk, arms, or face. Initial differential diagnoses included pigmented purpuric dermatoses (capillaritis), lichen planus, Flegel disease, and porokeratosis.

Diagnostic assessment

**Figure 3:**
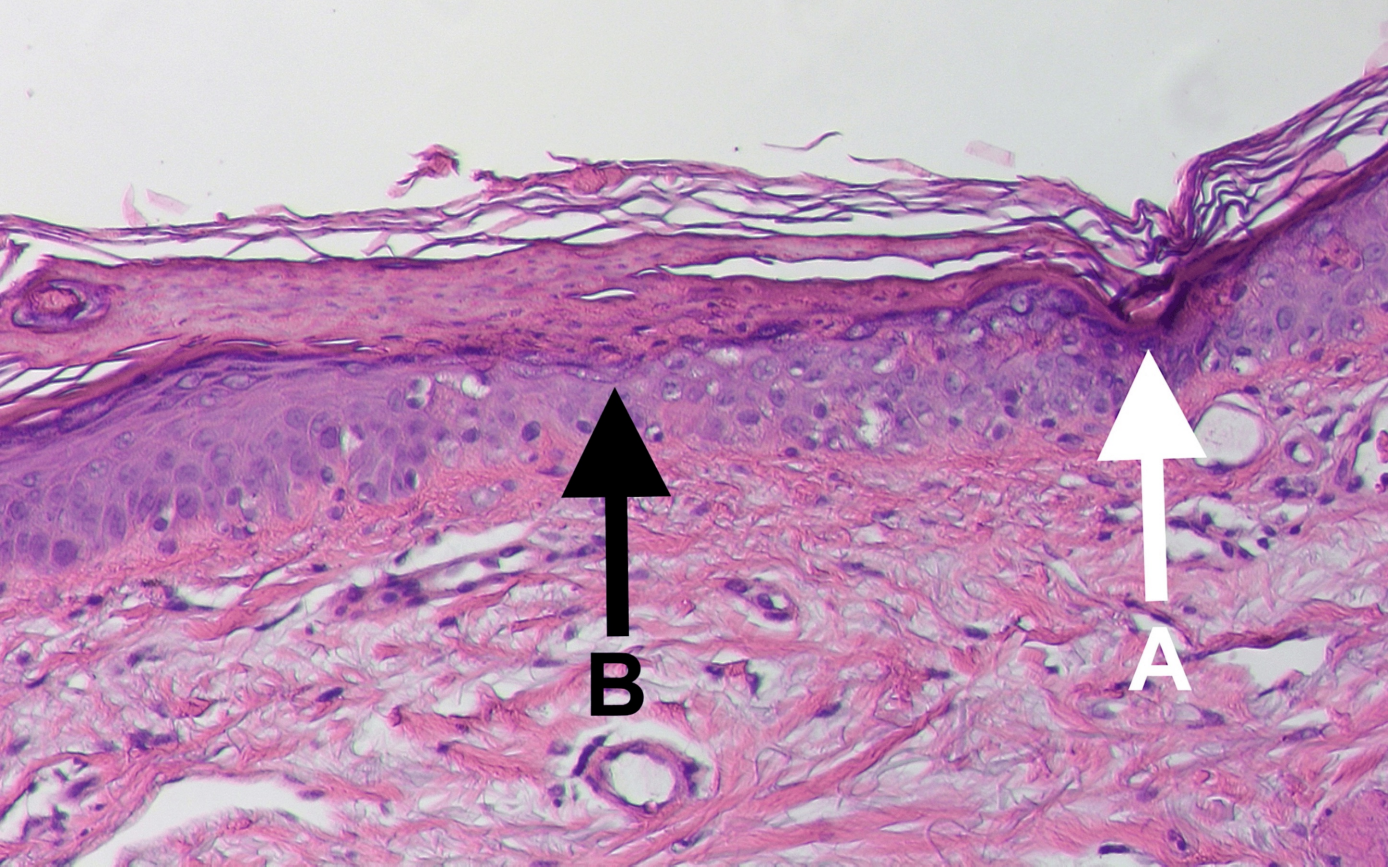
Histopathologic examination reveals features characteristic of porokeratosis, including a cornoid lamella (white arrow, A) and vacuolated, dyskeratotic keratinocytes originating from an invaginated, atrophic epidermis (black arrow, B). A sparse perivascular mononuclear infiltrate is also noted in the dermis.

Initial treatment with topical triamcinolone 0.1% cream was initiated, resulting in partial symptomatic relief; however, the papules continued to increase in number. A shave biopsy from the left shin was performed on April 11, 2025 (Figure 1). Histopathological examination revealed classic features of porokeratosis, including the presence of a cornoid lamella. Vacuolated and dyskeratotic keratinocytes were observed arising from an epidermal invagination within an atrophic epidermis (Figure 3). There was no evidence of viral cytopathic changes, granulomatous inflammation, or malignancy.

Therapeutic intervention

Given the confirmed diagnosis of EPPP and emerging evidence supporting the use of topical statin-based therapies, the patient was started on lovastatin 2% cream, applied twice daily to the lesions on the right lower leg. The left leg was left untreated to serve as an internal control for assessing treatment efficacy. The patient was counselled about the possibility of spontaneous resolution, which has been reported in up to 75% of cases over a six-month period [8].

Follow-up and outcomes

A comprehensive systemic evaluation was initiated given the reported associations between EPPP and underlying malignancy. The patient’s primary care physician conducted a thorough physical examination and ordered appropriate screening investigations, including complete blood count with differential (CBC), comprehensive metabolic panel (CMP), lactate dehydrogenase (LDH), chest radiography, and fecal occult blood testing. All evaluations were completed, and the results were within normal limits. Additionally, review of a colonoscopy performed two years earlier was also unremarkable.

As no controlled studies have compared the various treatment modalities for EPPP, no standardized guidelines currently exist for its management. Given that the patient was not significantly distressed by the eruption and that the condition is generally self-limiting, the benefit-to-risk assessment of available therapies supported a conservative approach. Topical agents with favorable safety profiles and minimal adverse effects were prioritized. A one-month trial of topical lovastatin 2% cream, applied twice daily to the right leg only, was initiated; however, there was no appreciable difference compared to the untreated left leg. No spontaneous improvement was observed, and new lesions continued to develop.

**Figure 4:**
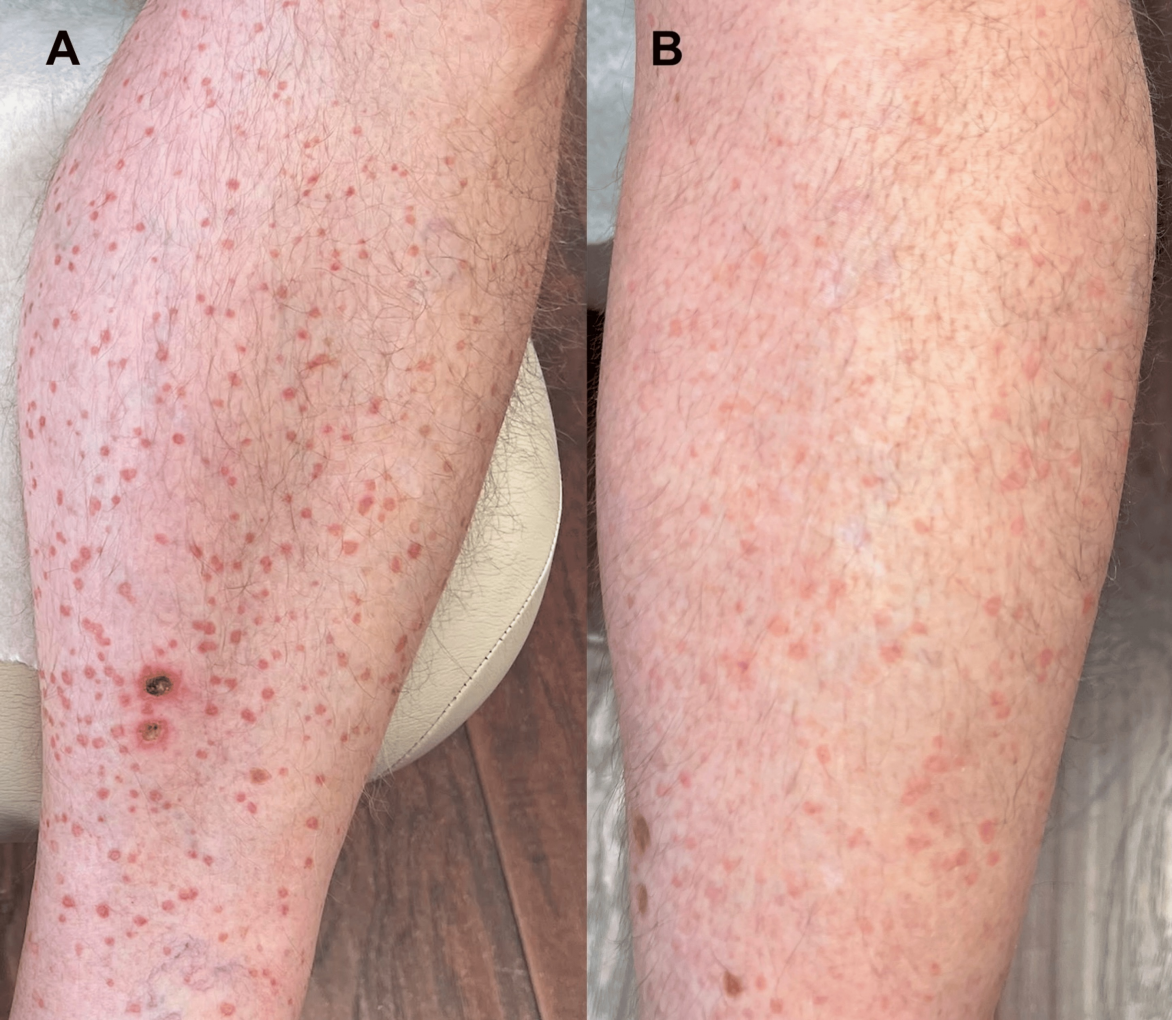
Therapeutic response of EPPP lesions to topical calcipotriene (A) Right leg showing initial presentation of EPPP lesions. (B) Right leg demonstrating marked reduction in the number and improved cosmesis of EPPP lesions following two months of topical calcipotriene 0.005% cream therapy twice daily. EPPP, eruptive pruritic papular porokeratosis

A topical calcipotriene 0.005% cream (Dovonex) was selected over alternative treatments such as 5-fluorouracil, imiquimod, and procedural interventions including laser ablation, cryotherapy, and dermabrasion, owing to its favorable safety profile, ease of application, and alignment with the patient’s preferences. The patient was instructed to apply the medication twice daily to the right leg only; however, during follow-up, it was noted that the patient had applied the cream to both legs consistently over the two-month period. Upon clinical reassessment, both legs demonstrated a marked reduction in lesion number and size, improved symptom control, enhanced cosmetic appearance, and overall patient satisfaction (Figure 4).

## Discussion

EPPP is a rare inflammatory variant of DSP. It is characterized by the abrupt onset of intensely pruritic papules, primarily affecting the extremities and trunk. EPPP typically occurs in older adults, with a median age of onset around 65 years, shows a slight male predominance, and may follow a chronic, relapsing course [2,3,8]. Histologically, EPPP is defined by the presence of cornoid lamellae - columns of parakeratotic cells overlying an absent granular layer - with possible additional features such as interface dermatitis and increased mononuclear inflammatory infiltrate, suggesting an underlying immunologic component [4,8].

The pathogenesis likely involves genetic and environmental interactions, including sun exposure, infections, pharmacologic agents, and immunosuppression [4,5,10-12]. Bednarek et al. and Yalcin et al. report eruptive porokeratosis in the context of corticosteroid use and diabetes, respectively, illustrating potential systemic contributors [10-13]. Goulding et al. observed rapid spontaneous resolution following drug cessation, suggesting reversible triggers in some cases [12]. This aligns with our patient’s course, where spontaneous regression was considered alongside therapeutic intervention.

Morgado-Carrasco et al. reviewed 32 cases of EPPP and proposed that the intense pruritus and sudden eruption distinguish EPPP clinically and immunopathologically from classic DSP [8]. Their review also noted that approximately 31.2% of cases were associated with underlying malignancies, predominantly gastrointestinal and hematologic cancers, highlighting the critical need for systemic evaluation upon diagnosis [8].

Our patient, a 77-year-old male, had no prior history of immunosuppression or malignancy. A colonoscopy performed two years prior was unremarkable, and additional systemic evaluation, including laboratory studies and imaging, did not reveal any occult neoplasm. Nevertheless, given the established association, ongoing age-appropriate surveillance is recommended. As the patient experienced minimal discomfort from the eruption and the condition is typically self-limiting, a conservative treatment strategy was pursued. Therapies with low-risk profiles and minimal adverse effects were prioritized. This included topical corticosteroids, topical statins, and topical calcipotriene.

Initial therapy with topical corticosteroids provided partial symptomatic relief but did not halt disease progression, consistent with prior reports indicating limited efficacy of conventional treatments [6,8]. Recent insights into the role of the mevalonate pathway have prompted the use of topical statins, which target the underlying metabolic dysregulation of keratinocyte proliferation. The proposed mechanism involves inhibition of 3-hydroxy-3-methyl-glutaryl-coenzyme A (HMG-CoA) reductase, thereby reducing the intracellular buildup of cytotoxic mevalonate metabolites [10]. Findings from a randomized clinical trial suggest that topical lovastatin 2% cream alone is both safe and effective, indicating that the addition of cholesterol may be unnecessary [11]. This supports its potential role as a primary treatment option for patients with DSAP [6]. Early use of statins has shown promise in porokeratosis subtypes, though further studies are needed to validate this approach [14,15]. Our use of topical lovastatin 2% ointment reflected this evolving pathogenesis-directed treatment strategy. Following the lack of clinical response to topical 2% lovastatin, topical calcipotriene 0.005% cream (Dovonex) was initiated due to its favorable safety profile and reported efficacy in other porokeratosis subtypes [16,17]. Calcipotriene, a vitamin D analog, exhibits anti-proliferative and immunomodulatory properties and may help normalize keratinocyte differentiation and modulate the local immune response. Case reports have described clinical improvement with calcipotriene in treatment-resistant porokeratosis, supporting its use in EPPP [16,17]. In this case, reassessment after a two-month trial demonstrated significant clinical improvement following the initiation of topical calcipotriene 0.005% cream. Our patient responded well to topical calcipotriene therapy, demonstrating significant clinical improvement in lesion number, size, symptom control, and cosmetic appearance. Given this favorable outcome, escalation to more aggressive therapies was not required. Older topical strategies such as 5-fluorouracil (5-FU) and imiquimod were reserved for later consideration due to their potential to induce significant local inflammatory reactions and a sequelae of scarring and hyper- or hypopigmentation [9]. Procedural interventions, including laser ablation therapies (erbium:YAG, CO₂, Q-switched Nd:YAG), were not considered due to their high cost, potential for post-procedural adverse effects such as erythema, hyperpigmentation, and edema, and the risk of lesion recurrence [18,19]. Dermabrasion was also deferred due to the extensive number of lesions and in accordance with the patient’s preference.

Had the patient demonstrated inadequate response to calcipotriene, a shared decision-making discussion would have been initiated to explore alternative topical therapies, such as 5-FU or imiquimod. In cases that are severe or refractory, emerging systemic treatments, including JAK inhibitors and omalizumab, are currently under investigation and may offer additional therapeutic options in the future [6,7].

Spontaneous resolution is a recognized outcome in EPPP, typically occurring within six months to two years following onset [8]. While Morgado-Carrasco et al. noted that treatment responses are often modest and that most cases eventually resolve without intervention, this natural course is generally prolonged. In contrast, our patient demonstrated marked clinical improvement within a two-month period following initiation of topical calcipotriene, a timeframe that is inconsistent with spontaneous remission. Given the rapid and bilateral reduction in lesion number and size, as well as improvement in symptoms and cosmesis, the therapeutic response is more plausibly attributed to the pharmacologic effects of calcipotriene rather than to the natural disease course. This case therefore supports the potential utility of early low-risk intervention in select patients, particularly when conservative observation may delay meaningful symptom relief and quality-of-life improvement.

Table 1 summarizes the clinical and histopathological features of our patient in comparison to previously reported EPPP cases, as reviewed by Morgado-Carrasco et al. [8]. EPPP typically presents in older adults, with a median age of onset around 65 years and a slight male predominance, features consistent with our 77-year-old male patient. He exhibited the classic presentation of a sudden eruption of intensely pruritic papules on the lower extremities. Histologically, findings such as a cornoid lamella and perivascular mononuclear infiltrate were observed, aligning with diagnostic features described in the literature. While Morgado-Carrasco et al. reported an association with internal malignancies in approximately one-third of cases, most commonly gastrointestinal or hematologic, no malignancy has been identified in our patient to date.

**Table 1:** Comparison of clinical features between the present case and reported EPPP Cases Our patient’s presentation was consistent with prior EPPP cases in terms of age, sex, sudden onset of pruritic papules, and histologic findings of a cornoid lamella with perivascular inflammation. Notably, while Morgado-Carrasco et al. [8] documented internal malignancies in about one-third of cases and prior dermatoses in over half, no malignancy or antecedent dermatosis was identified in our patient, highlighting the heterogeneity of clinical outcomes in EPPP. EPPP, eruptive pruritic papular porokeratosis

Clinical Feature	Present Case	Morgado-Carrasco et al. (2020) [8]
Age	77 years	Median 66 years (range 13–84)
Sex	Male	59.3% male
Lesion distribution	Lower extremities	Extremities and back
Pruritus	Present	Present in all cases
Histopathology	Cornoid lamellae, vacuolated, dyskeratotic keratinocytes, scant perivascular mononuclear infiltrate	Cornoid lamellae in all cases; inflammatory infiltrate in 53%
Associated neoplasms	None identified at time of writing	31.2% of cases
Associated viral infections	None reported	6 cases reported
Previous dermatoses	None reported	Reported in 14 cases (52%)
Treatment response	Partial improvement with topical corticosteroid. No improvement with topical statin cream after 1 month. Significant clinical improvement with topical calcipotriene cream after 2 months.	Poor in most cases
Spontaneous resolution	Follow-up ongoing	75% resolved spontaneously
Time to resolution	Follow-up ongoing	Median 6 months (range 1–24 months)

This rare case reinforces the importance of recognizing EPPP as a distinct clinical entity, prompting appropriate malignancy workup and consideration of emerging therapeutic options such as topical statins and vitamin D analogs. Management should be individualized, accounting for cosmetic and functional factors as well as patient preferences. The chronic, treatment-resistant nature of our patient's condition underscores the variability in clinical course and therapeutic response seen in EPPP. Continued documentation of cases like this is essential to advancing our understanding of this rare condition and guiding evidence-based treatment strategies.

## Conclusions

EPPP is a rare and distinct inflammatory variant of DSP that often presents a diagnostic and therapeutic challenge. This case underscores the importance of histopathologic confirmation and systemic evaluation. With most cases resolving spontaneously or with conservative management, treatment may be tailored to symptom burden. Emerging therapies, such as calcipotriene, represent promising low-risk treatment options for EPPP, though further reporting and investigation are necessary to clarify their long-term efficacy and place in management. Continued documentation of EPPP cases will improve understanding of its pathogenesis, guide effective therapies, and refine approaches to malignancy screening.
